# Limited Scleroderma-Induced Pulmonary Arterial Hypertension Resulting in Impaired Postoperative Respiratory Function

**DOI:** 10.7759/cureus.13742

**Published:** 2021-03-06

**Authors:** Farhan A Shah, Nathan Mahler, Michalla Braford, Nelson Greene

**Affiliations:** 1 Internal Medicine, Lewis Gale Medical Center, Salem, USA; 2 Internal Medicine, Edward Via College of Osteopathic Medicine, Blacksburg, USA; 3 Pulmonary and Critical Care, Lewis Gale Medical Center, Salem, USA

**Keywords:** scleroderma, pulmonary arterial hypertension

## Abstract

Limited scleroderma falls under the umbrella of systemic sclerosis, an autoimmune disease that presents with multiorgan dysfunction that includes pulmonary arterial hypertension. We examine a case of pulmonary arterial hypertension in an elderly nonsmoker with a history of limited scleroderma.

The patient presented with abdominal tenderness and was diagnosed with a sigmoid colonic stricture. She underwent laparoscopic bowel resection. During and after her surgery, she suffered from worsening respiratory function and decompensated, developing a large pleural effusion that led to a thoracentesis and a prolonged hospital course.

Patients with scleroderma can develop acute symptoms involving several organ systems, including the colonic tract and lungs, as seen in our patient. A thorough workup and continuous close management and monitoring are necessary to avoid further complications in these patients, especially in the postoperative period.

## Introduction

Systemic sclerosis (SSc) is a multifaceted autoimmune condition that affects several organ systems through fibrosis, vasculopathy, and immune activation. Limited cutaneous SSc (lcSSc) or limited scleroderma is a subset of systemic sclerosis with involvement restricted to the skin, with or without facial involvement, and the distal limbs below the knee-elbow. According to the 6th World Symposium on Pulmonary Hypertension in 2018, the updated definition of pulmonary arterial hypertension (PAH) is now a mean pulmonary artery pressure (mPAP) >20 mmHg. PAH and interstitial lung disease cause the most deaths due to systemic sclerosis [1]. We present a unique case of pulmonary arterial hypertension in a 78-year-old female nonsmoker with a history of limited scleroderma.

## Case presentation

**Figure 1:**
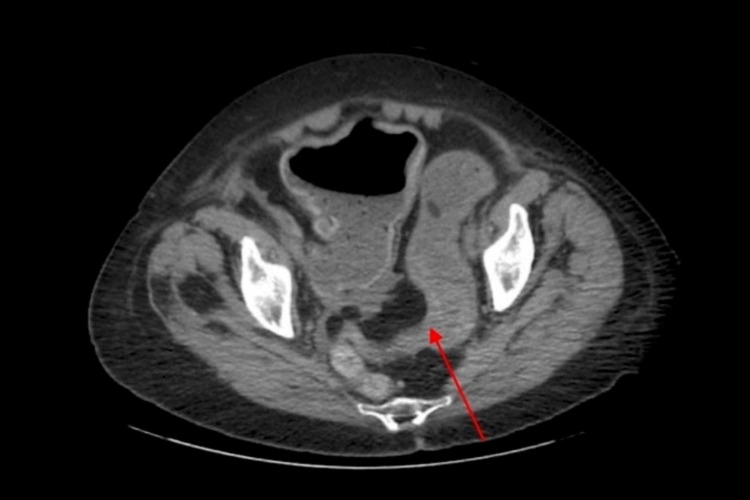
CT abdomen and pelvis revealed diffuse distention of the colon leading up to an abrupt bird beak transition to a collapsed colonic segment at the mid to distal sigmoid (red arrow)

The patient is a 78-year-old Caucasian female nonsmoker with a past medical history of limited scleroderma (anti-centromere antibody positive), PAH, gastroesophageal reflux disease, hypothyroidism, and atrial fibrillation. She initially presented to a neighboring hospital complaining of a four-day history of abdominal tenderness. She described it as diffuse, constant, aching, and dull in nature, non-radiating, worse with movement, and relieved with rest. She stated that she had not had a bowel movement in two days. Physical examination revealed diffuse tenderness to palpation, without guarding or rebound tenderness. Initial vitals were abnormal for hypertension of 156/82 and mild tachycardia of 102 beats/minute. CT abdomen and pelvis without contrast revealed diffuse distention of the colon, leading up to an abrupt bird beak transition to a collapsed colonic segment at the mid to distal sigmoid (Figure 1). The patient developed pneumonia with associated dyspnea requiring oxygen supplementation via nasal cannula and atrial fibrillation with rapid ventricular response. She was then started on antibiotics, placed on a diltiazem infusion, and was transferred to our facility’s intensive care unit. Subsequent colonoscopy confirmed a sigmoid colon stricture, and a decompression tube was placed. 

**Figure 2:**
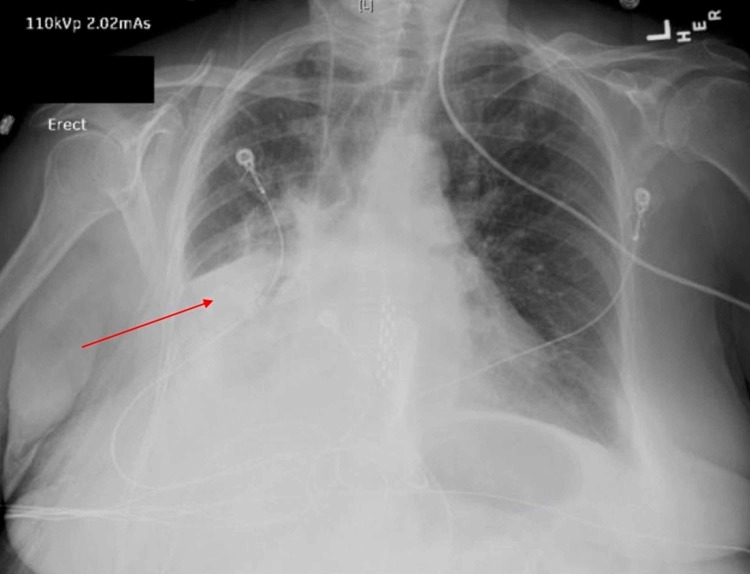
Chest radiograph revealed a large right-sided pleural effusion (red arrow)

The patient underwent planned laparoscopic bowel resection. However, due to her persistent hypoxia and respiratory failure while under anesthesia, she was could not tolerate the procedure and was given a colostomy bag instead of resectioning of the sigmoid colon. The patient underwent difficult extubation, subsequently resulting in hypoxia, and was placed on bilevel positive airway pressure (BIPAP). A chest radiograph revealed a large right-sided pleural effusion (Figure 2).

She underwent a thoracentesis that removed 925 cc of serous fluid, sent for analysis and pathology (Table 1). Cytopathology report of the pleural fluid revealed markedly reactive mesothelial cells and macrophages, indicative of acute chronic inflammation. Her oxygenation improved, allowing the patient to be discharged home without any further complications.

**Table 1:** Pleural fluid analysis WBC: white blood cells, RBC: red blood cells, LDH: lactate dehydrogenase

Pleural fluid characteristics	Pleural fluid analysis results
Color	Straw
Appearance	Hazy
Pleural WBC	676 /μL
Pleural RBC	2000 /μL
Pleural Polynuclear WBC	26%
Pleural Lymphocytes	65%
Pleural Monocytes	6%
Pleural Mesothelial cells	3%
Pleural Albumin	1.2 IU/L
Pleural LDH	82 IU/L
Pleural Glucose	105 mg/dL

The patient’s chronic pulmonary hypertension in combination with acute pneumonia resulted in an impaired surgical outcome. The patient had an echocardiogram the previous year revealing a right ventricular systolic pressure of 51 mmHg with a normal left ventricular systolic function. A subsequent right heart catheterization showed a mean pulmonary artery pressure of 30 mmHg, and a pulmonary capillary wedge pressure (PCWP) of 16 mmHg. Pulmonary function tests (PFTs) showed a normal diffusion capacity of the lungs for carbon monoxide (DLCO) with a forced expiratory volume in one second (FEV1)/forced vital capacity (FVC) ratio of 78%. After other etiologies were ruled out, her pulmonary hypertension (PH) was classified as World Health Organization (WHO) functional class I secondary to limited scleroderma. 

## Discussion

Scleroderma is an autoimmune disease causing inflammatory changes in multiple tissues, including the lungs, which may manifest itself in various forms, such as PAH, and interstitial lung disease [2]. Systemic sclerosis-induced PAH (SSc-PAH) was previously defined as a mean pulmonary artery pressure (mPAP) >25 mmHg on right heart catheterization (RHC) and PCWP ≤15 mmHg, without any evidence of any significant pulmonary parenchymal disease. However, the 6th World Symposium on Pulmonary Hypertension in 2018 now defined PAH as mPAP >20 mmHg [1]. There are five different groups of pulmonary hypertension (PH) based on its various causes. The groups are defined by the WHO and are known as the PH WHO Groups or the WHO Functional Classes. Scleroderma may cause extensive fibrosis in pulmonary arterioles, causing group 1 PH, or PAH (the most common form of PH patients with sclerosis). This type of PAH is similar to idiopathic PAH in individuals with no rheumatologic disease. Scleroderma causing significant myocardial damage can lead to group 2 PH. Interstitial or restrictive lung disease is also common, leading to group 3 PH. Group 4 PH is due to pulmonary artery obstructions secondary to chronic thromboembolic PH. Lastly, group 5 PH is due to an unclear or multifactorial mechanism, for example hematologic disorders [1].

PAH is likely to present in 10 to 15% of patients with systemic scleroderma, with an even higher percentage in patients with limited cutaneous scleroderma [2,3]. However, previous studies have excluded patients with concurrent pulmonary fibrosis. Increased mortality is partially attributed to the difficulty in identifying PAH until disease progression is more severe [4]. Risk factors of developing scleroderma-induced PAH include disease duration or increased age at the time of diagnosis of systemic sclerosis [5], the presence of limited cutaneous SSc [6], a low DLCO [7], the presence of the limited subtype of SSc, anti-centromere antibody (ACA)-positive, and the presence of telangiectasia [8]. Risk stratification is necessary to help evaluate patients' mortality rates with PAH while considering factors that include: signs of right heart failure, WHO Functional Class, and sex, as males are considered more prone to developing PAH than females [9,10]. The European League Against Rheumatism (EULAR) Scleroderma Trials and Research database found isolated PAH associated with 9.2% of limited scleroderma compared to 5.8% of diffuse scleroderma patients [11]. The prevalence of systemic sclerosis-induced PAH overall has been found to range between 13 to 35% when diagnosed through an echocardiogram and 8 to 12% when diagnosed through RHC. However, it is critical to note that the relatively recent change in the definition of PAH to include an mPAP >20 will most likely increase the prevalence of SSc-PAH [10]. 

The pathophysiology of SSc-PAH is unclear. Current understanding involves injury to the vascular endothelium, inflammation, angiogenesis, and subsequent arterial obliteration. This process results in fibrosis which leads to increased pulmonary arterial pressures [11]. Specifically, isolated PAH is more common in limited scleroderma and is associated with increased numbers of anti-fibrillarin (anti-U3-RNP) antibodies. Anti-fibrillarin antibodies are thought to influence pathogenesis through upregulation of adhesion molecules and class II histocompatibility molecules located on pulmonary artery endothelial cells. Upregulation is associated with an increased risk of inflammatory vasculopathy [12].

Screening for PAH in patients with scleroderma is essential for identifying the disease early to be treated. PAH is the leading cause of mortality in patients with scleroderma, with a two-year survival reported at 40% in individuals with isolated PAH compared to an 80% survival without PAH [13]. There are multiple approaches to screening based on organization practice guidelines, but most clinicians agree that all patients with scleroderma should have a baseline set of PFTs and be screened for symptoms of lung disease at each visit [8]. An increased probability of developing PAH occurs with a decrease in the DLCO and an FVC/DLCO ratio (FVC/DLCO >1.6) [10]. The gold standard for diagnosing PAH is right heart catheterization (RHC). However, RHC carries risks due to complications stemming from its inherent invasiveness and increased hospital costs [10]. An echocardiogram is a non-invasive alternative to the RHC. However, it is limited by its operator-dependent nature and its indirect estimation of pulmonary arterial pressures (PAP) from right ventricular pressure (RVP) and tricuspid regurgitation velocity (TRV) (which can be limited in accuracy due to body habitus). The 2015 European Society of Cardiology/European Respiratory Society (ESC/ERS) guidelines recommend that clinicians perform initial screening upon systemic scleroderma diagnosis by echocardiogram and subsequent RHC if there is a positive screen on the echocardiogram [10]. Despite a normal DLCO, this patient had abnormal findings on an echocardiogram. Therefore, a right heart catheterization, which is the gold standard for diagnosing PAH, was performed and confirmed its presence.

Additionally, four primary systemic sclerosis-related antibodies are known to be associated with an increased risk of PAH: anti-centromere, anti-Th/To, anti-U1 ribonucleoprotein (RNP), and anti-U3 RNP. Patients found to have at least one of these four antibodies or a nucleolar pattern anti-nuclear antibody (ANA) should be carefully monitored for signs of developing PAH and should undergo routine clinical screening [10].

According to the 2003 World Symposium on Pulmonary Hypertension guidelines, treatment of the WHO group 1 PH has primarily consisted of pharmacologic agents targeting the nitric oxide, endothelin, as seen in our patient and prostaglandin pathways. General preventive treatment measures include avoiding pregnancy, influenza, and pneumococcal vaccines, and avoiding general anesthesia, and using epidural anesthesia, if it is a viable alternative. Supportive therapy includes utilizing diuretics for right heart failure and fluid overload, chronic oxygen support if partial pressure of oxygen (PO2) is less than 60 mmHg, calcium channel blockers such as nifedipine and diltiazem, endothelin receptor antagonists such as ambrisentan and bosentan, phosphodiesterase-5 inhibitors such as sildenafil and tadalafil, and, when possible, avoiding angiotensin-converting enzyme (ACE) inhibitors, angiotensin receptor blockers (ARBs), and beta-blockers. Patients diagnosed with PAH must be offered an early referral to tertiary care PH centers, where they can undergo a multidisciplinary treatment regimen involving coordination between pulmonologists, cardiologists, and general internists [9]. 

## Conclusions

Systemic or limited scleroderma-induced pulmonary arterial hypertension is a devastating consequence of the systemic manifestations of sclerosis. Careful and diligent follow-up after a patient is diagnosed is necessary to avoid further respiratory decompensation. Clinicians should be aware of how tenuous a patient’s respiratory function can be in the postoperative stage if they have a history of pulmonary arterial hypertension and monitor them accordingly.

## References

[1] Simonneau G, Montani D, Celermajer DS (2019). Haemodynamic definitions and updated clinical classification of pulmonary hypertension. Eur Respir J.

[2] Muangchan C, Canadian Scleroderma Research Group, Baron M, Pope J (2013). The 15% rule in scleroderma: the frequency of severe organ complications in systemic sclerosis. A systematic review. J Rheumatol.

[3] Hunzelmann N, Genth E, Krieg T (2008). The registry of the German Network for Systemic Scleroderma: frequency of disease subsets and patterns of organ involvement. Rheumatology (Oxford).

[4] Mukerjee D, St George D, Coleiro B (2003). Prevalence and outcome in systemic sclerosis associated pulmonary arterial hypertension: application of a registry approach. Ann Rheum Dis.

[5] Avouac J, Airò P, Meune C (2010). Prevalence of pulmonary hypertension in systemic sclerosis in European Caucasians and metaanalysis of 5 studies. J Rheumatol.

[6] Cox SR, Walker JG, Coleman M (2005). Isolated pulmonary hypertension in scleroderma. Intern Med J.

[7] Ferri C, Valentini G, Cozzi F (2002). Systemic sclerosis: demographic, clinical, and serologic features and survival in 1,012 Italian patients. Medicine (Baltimore).

[8] Hao Y, Thakkar V, Stevens W (2015). A comparison of the predictive accuracy of three screening models for pulmonary arterial hypertension in systemic sclerosis. Arthritis Res Ther.

[9] Yaghi S, Novikov A, Trandafirescu T (2020). Clinical update on pulmonary hypertension. J Investig Med.

[10] Saygin D, Domsic RT (2019). Pulmonary arterial hypertension in systemic sclerosis: challenges in diagnosis, screening and treatment. Open Access Rheumatol.

[11] Solomon JJ, Olson AL, Fischer A, Bull T, Brown KK, Raghu G (2013). Scleroderma lung disease. Eur Respir Rev.

[12] Okawa-Takatsuji M, Aotsuka S, Fujinami M, Uwatoko S, Kinoshita M, Sumiya M (1999). Up-regulation of intercellular adhesion molecule-1 (ICAM-1), endothelial leukocyte adhesion molecule-1 (ELAM-1) and class II MHC molecules on pulmonary artery endothelial cells by antibodies against U1-ribonucleoprotein. Clin Exp Immunol.

[13] Stupi AM, Steen VD, Owens GR, Barnes EL, Rodnan GP, Medsger TA Jr (1986). Pulmonary hypertension in the CREST syndrome variant of systemic sclerosis. Arthritis Rheum.

